# Understanding the Link Between Burnout and Sub-Optimal Care: Why Should Healthcare Education Be Interested in Employee Silence?

**DOI:** 10.3389/fpsyt.2022.818393

**Published:** 2022-03-31

**Authors:** Anthony Montgomery, Olga Lainidi

**Affiliations:** Department of Educational and Social Policy, University of Macedonia, Thessaloniki, Greece

**Keywords:** employee silence, burnout, Quality of care (QoC), healthcare education and training, wellbeing

## Abstract

Evidence on the association of burnout with objective indicators of performance is scarce in healthcare. In parallel, healthcare professionals ameliorate the short-term impact of burnout by prioritizing some tasks over others. The phenomenon of employee silence can help us understand the evolution of how culture is molded toward the prioritization of some tasks over others, and how this contributes to burnout. Silence in healthcare has been associated with concealing errors, reduced patient safety, and covering up errors made by others. Conversely, there is evidence that in organizations where employees are encouraged to speak up about concerns, and where concerns are responded to appropriately, better patient outcomes such as improved patient safety and patient experience occur. Interventions to promote “speaking-up” in healthcare have not been successful and are rooted in a professional culture that does not promote speaking out. In this paper, we review the evidence that exists within healthcare to argue why healthcare education should be interested in employee silence, and how silence is a key factor in understanding how burnout develops and impacts quality of care. The following key questions have been addressed; how employee silence evolves during medical education, how is silence maintained after graduation, and how can leadership style contribute to silence in healthcare. The impact of withholding information on healthcare professional burnout, patient safety and quality of care is significant. The paper concludes with a suggested future research agenda and additional recommendations.

## Introduction

Burnout is accepted as a significant problem in healthcare, and there is a plethora of research to demonstrate the links with patient safety and quality of care. However, evidence on the association of burnout with objective indicators of performance (as opposed to self-report) is scarce in all occupations, including healthcare (1, 2). But, the research that does exist indicates an important relationship between burnout and sub-optimal care. For example, intensive care units in which staff reported high levels of emotional exhaustion had higher patient standardized mortality ratios, even after objective unit characteristics such as workload had been controlled for Welp et al. (3). Thus, we have a challenge in identifying the links that connect burnout and performance in healthcare (4).

An under-researched area in the literature is the way that healthcare professionals maintain performance during stressful conditions. For example, there is some evidence that even when staff lacks mental or physical energy (5) they use “performance protection” strategies to maintain high priority clinical tasks and neglect low priority secondary tasks (such as reassuring patients) (6). The simplest example of this is the existence and frequent use of “work arounds” in healthcare, whereby staff develop creative solutions to resource and/or staff shortages (7). The drive for healthcare professionals to “keep going” and “get the job done” has a dark side referred to as pathological altruism (8), which refers to behaviors that attempt to promote the welfare of another but can have pernicious long-term consequences for the care giver. Healthcare exploits the professional ethic of healthcare professionals which results in a form of dysfunctional professionalism that support maladaptive healthcare structures in education and practice (9). The gap between what our healthcare workers would need to balance maintaining quality of care with their wellbeing and the reality of their day-to-day experience is significant—as is evidenced by increasing levels of burnout. Self-care equals safe care, but that is not happening as burnout and the associated mental health problems are not dealt with until a tipping point is reached.

The phenomenon of employee silence in healthcare can help us to understand how culture is molded toward the prioritization of some tasks over others. Indeed, a common phenomenon among healthcare staff is a feeling that they are unable to share their concerns, and their managers are anxious about even seeking them or having honest informal conversations. Thus silence is the result of such “protective hesitancy” as both may not feel it is “psychologically safe” to have such discussions (10, 11). Understanding what healthcare workers consider to be a priority can be understood via what they speak-up about and keep silent about at work. Remaining silent about important issues is a complex phenomenon in healthcare that is rooted in early educational experiences and career development. Silence is connected with low levels of psychological safety (12), which in turn is connected with burnout and poorer care (13, 14). From this perspective, burnout and sub-optimal care are symptoms of a dysfunctional system. Such systems are difficult to analyze directly, but employee silence provides an important bridge into how healthcare workers make sense of their work via what they choose to speak-up about and what they remain silent about. Moreover, viewing this phenomenon through the educational journey of healthcare professionals delineates the evolution of what is acceptable/not acceptable to discuss which is directly linked to employee wellbeing, patient safety and quality of care.

In this paper, we review the evidence that exists within healthcare to argue why healthcare education should be interested in employee silence. We will focus on the experience of physicians, given their pivotal role in healthcare delivery, but the conclusions we reach are relevant for healthcare education generally. The following key questions have been addressed; how employee silence evolves during medical education, how is silence maintained after graduation, and how can leadership style contribute to silence in healthcare. The paper concludes with the future research agenda and some recommendations.

## What Is Employee Silence and Why Is it Important?

Employee silence denotes the withholding of genuine expressions about employees’ evaluations of personal, social, and/or organizational circumstances at work to persons who are capable of effecting change at work (15). Employee silence in healthcare falls into two broad categories; voluntary and involuntary forms of silence. Silence in healthcare can take a number of forms that include being silent about patient safety concerns and covering up errors (16–18), ethical issues (19), discrimination issues (20), inappropriate behavior (21, 22), neglected care (23), and lack of resources (24). Conversely, there is evidence that in organizations where employees are encouraged to speak up about concerns, and where concerns are responded to appropriately, better patient outcomes such as improved patient safety and patient experience occur (25). The need for us to address employee silence in healthcare has been highlighted by a recent systematic review which concluded that speaking-up interventions in healthcare are largely ineffective, due to a global pervasiveness and dominance of professional cultures that are inimical to speaking-up interventions (26).

**FIGURE 1:**
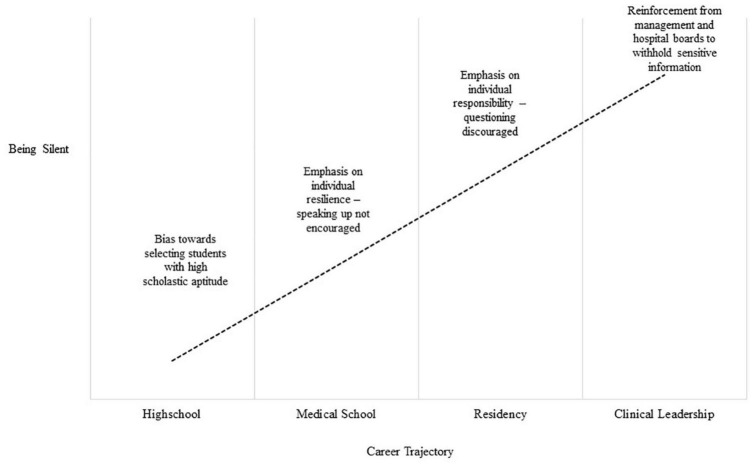
Examples of forces contributing to silence.

In the following, we will argue that the phenomenon of employee silence has its roots in medical education systems that reflect the prevailing values within society that valorize competitiveness and status. Prompts toward silence and the need to protect the in-group (i.e., physicians) starts in medical school and is entwined in the continuous forms of education that healthcare professionals attend to during their career. Physicians are educated to be clinicians first, and their role as a leader, team member, or manager is secondary (27). This system results in a formal culture that values professionalism, but a hidden one that valorizes performance and competitiveness above collaboration (28, 29). Figure 1 highlights examples of forces that contribute to employee silence during the career of a physician. In the first part of the paper, we will review the evidence indicating how employee silence evolves and is maintained. In the second part of the paper, we will identify avenues for future research and make recommendations as to what can be done to address employee silence.

## How Does Employee Silence Evolve During Medical Education?

There is significant evidence that medical training is plagued by difficulties with the delivery of undesirable information regarding the assessment of students (30), which represents a lost opportunity in terms of modeling the sharing of information. A systematic review on selection methods used in medical education highlights the fact that outcome measures used to evaluate selection methods most often focus on indicators of attainment and maximal performance (e.g., medical school achievements, performance in licensure examinations) rather than indicators relating more directly to clinical practice (31). This begs the question as to whether healthcare education is building a culture of performance first; where individual wellbeing and asking difficult questions is far down the list of priorities (8, 9). The fact that such high percentages of physicians consistently report symptoms of burnout (32) suggests that there is a significant problems with the job and its ability to adequately support individuals to meet its demands (33), and this problem will not be ameliorated if the response of healthcare organizations is to focus predominately on individual-focused solutions (e.g., extended leave, mediation, psychotherapy) (34). A “performance first” culture does not encourage speaking-up. For example, a BMJ blog written by two new United Kingdom medical students argues that there is unhealthy focus on individual resilience which results in them compensating for a flawed system, and sounds more like compliance than resilience. The students conclude by arguing that junior doctors need to be empowered to build more resilient systems, by whistleblowing, advocating, and speaking out against wrong (35).

The profile of the “average” medical student is someone with high scholastic performance and high levels of adaptive perfectionism (36). Unfortunately, the “average” medical student commonly reports symptoms of depression (37) and burnout (38) as a consequence of the demands of medical training. The inhibition of emotion that results from having to remain silent can have a huge psychological toll (39). If junior healthcare staff believe that certain forms of silence based on loyalty or “not breaking ranks” is expected of them, they run the risk of underestimating the impact on their own wellbeing. Moreover, staff can carry this rumination home making recovery from work less effective (40)—with medical education being the first exposure to this phenomenon. The challenge for medical education is to avoid promoting the value of self-sacrifice as the characteristic of a good healthcare professional, as the ideal of sacrifice can dovetail in a dysfunctional way with being loyal, and not speaking up.

The significant literature on the hidden curriculum suggests that the induction period for many young physicians is characterized by a toxic performance culture, whereby adversity is viewed as “character building” and emotional repression is valorized (41, 42). Moreover, evidence indicates that medical students report inaction in the face of emotionally challenging situations (43, 44), and dysfunctional emotion regulation strategies can be a risk factor for burnout (i.e., emotional exhaustion, and cynicism) (45). Ultimately, it appears that young physicians learn early on that certain dysfunctional behaviors are valued (e.g., working long hours without appropriate breaks as an indicator of “commitment”).

## How Is Silence Maintained Post Residency?

As young physicians begin their careers, there are many prompts from their environments that reinforce the tendency toward withholding information. As already noted, the use of “work arounds” is frequent in healthcare. Work arounds could be viewed as an organic response to acquiescent silence (i.e., apathy)—resulting from a belief that problems need to be “worked around” because change is not forthcoming. Ultimately, such adaptations may not be spoken about because they grow out of the situation the staff is in, and they are seen as natural necessities rather than as true innovations. Thus, there is acceptance that being “silent” about gaps in care is practical and solution-focused. Congruently, open discussions of medical errors are sensitive due to the legal ramifications of sharing information that may identify the malpractice of a coworker, thus limited sharing of such information can be viewed as critical to maintaining work team cohesion.

Policies or interventions to give healthcare professionals opportunities to voice may not effectively reduce silence, and therefore fail to reduce burnout, if employees still withhold issues they do not feel comfortable sharing (46). Researchers and practitioners cannot assume that physicians who frequently speak up are not withholding other issues (47). For example, in 2016 the UK National Health Service (NHS) introduced the “Freedom to Speak Up Guardian” (FTSUG) role, with the objective of improvements in the way staff concerns were handled and responded to (especially with regard to patient safety). Interestingly, thousands of NHS staff have spoken up to FTSUGs, but the majority of concerns raised were about bullying and harassment behaviors by colleagues, rather than direct patient safety concerns (48). The challenge for healthcare education is enable physicians to speak about professional behavior more directly. Additionally, there is a secondary issue as to whether bullying/harassment is a “strategy” to encourage employee silence. For example, in the paper of Edwards et al. a step-by-step analysis of the case of “Dr. Death” at the Bundaberg Hospital in Australia revealed that numerous allegations of harassment and bullying were filed prior to the official inquiry starting (49). The inquiry concluded that 13 patients died due to negligence, and highlighted how harassment and bullying behaviors were used to intimidate junior staff into silence.

Thus, the challenge for continuous professional development (CPD) is to understand the processes that result in organizations consisting of people who promote silence as a norm, and understand how CPD can be used to equip healthcare professionals with tools to promote the appropriate sharing of information. A good place to start is to explore how CPD can contribute to building an inclusive workplace, meaning workplaces and teams where the differences and uniqueness that staff bring are valued, as organizations are more likely to be “psychologically safe” workplaces where staff feel confident in expressing their true selves, raising concerns and admitting mistakes without fear of being unfairly judged (50).

## How Can Leadership Style Contribute to Silence in Healthcare?

Healthcare professionals often fear blame, loss of jobs, legal issues or breaking the hierarchy as they hesitate to speak about errors and transgressions. There is considerable anecdotal evidence in healthcare that the silence norm is top-down. For example, the chairs of medicine and surgery departments report it is common for faculty not raise or talk about important problems (51). What are the types of behaviors modeled by leadership and line-management that promote silence? The evidence indicates that toxic supervisors, who avoid adopting subordinate’s ideas, can lead employees to be more silent (52). If the aforementioned behaviors are characteristic of clinical leaders, they will feed into the group climate of the unit and/or the department. Large power discrepancies are ingrained in medical culture and adversely affect “low power” members’ perception regarding their willingness to speak up, which inhibits productive communication (24, 53).

We can view employee silence as an “appropriate” reaction to certain leadership styles, group dynamics and/or as a vehicle for participation in the organization rather than a withdrawal from it. Given the widespread nature of employee silence in the health sector, the complexity of medical healthcare provision and the augmented stress that characterizes healthcare professions, we should also consider that the extent to which employee silence can be identified in the healthcare sector might also be a type of participative group climate (or intragroup norm), in particular if we take into account all plausible motivations for being silent about medical errors and mental health issues faced by healthcare professionals or other problems in the sector. Group climate is characterized by shared employee perceptions and it has been suggested through research findings that group-level perceptions related to psychological safety can predict individual voice behaviors (54). In this case, if speaking up is perceived as a threat to the psychological safety on a group level, then the tendency to be silent—or speak up less—as an individual, could be an expression of a participative climate.

## Charting the Future Research Agenda

**FIGURE 2:**
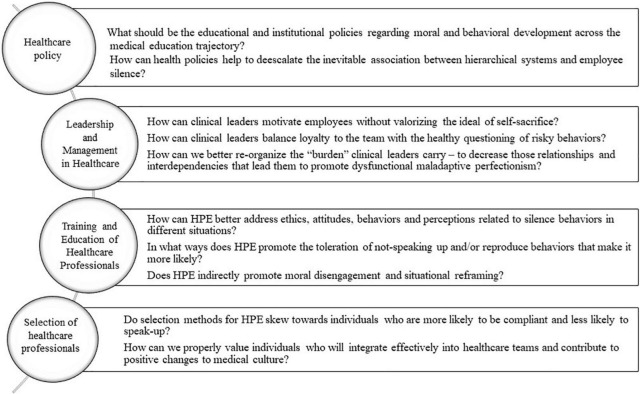
Research agenda for addressing silence in health professionals education (HPE).

Future research in the field of heath professional education needs to meaningfully reflect the influence of individual factors, group factors, and context on learning, performance, and wellbeing. Education is an *organized* activity, and shouldn’t be treated as a value-free preparation for professional life. Issues that are related to group dynamics, like collaboration, competitiveness, leadership, effectiveness, decision making, organizational culture and development—which are the focus of the medical profession—should also be the focus of medical education. In this context, behaviors that are considered individual choices—e.g., to speak up or to remain silent—can be also studied as “organizational phenomena,” and in healthcare some of those organizational phenomena are very likely to also have their roots in education. Medical education needs to move from a focus on individual attainment to collective effectiveness, which is more likely to promote individual wellbeing and thus patient safety. Figure 2 outlines suggested questions that future research needs to address.

## Further Thoughts and Recommendations

Evidence from industries outside of healthcare is instructive. For example, Shaukat and Khurshid (55) found that burnout mediated the relationship between employee silence and employee performance, leading to withdrawal behaviors and turnover intention among telecom engineers. Thus, if supervisors do not encourage employees to share their work-related concerns, silence functions as a workplace stressor that starts a loss of resources process, in agreement with the resource depletion principle predicted by the Conservation of Resources (COR) theory (56). In this context, one could argue that the more pervasive silence is, the more intense is the effect on the experience of burnout, specifically emotional exhaustion (57).

The concept of organizational memory is very useful. For example, research on the dark side of policing highlights the norms that can support police silence and which are integrated into organizational memory (58). Such norms include; not “ratting” on another officer, not implicating your colleagues if you’re caught doing something, not interfering with the activities of other police offices, not trusting new people until they have been socialized into the norms, and not volunteering information about any event that could implicate a colleague. The important point is that such behaviors are learned early in the educational experiences. Not surprisingly, corrupt decisions that result in positive outcomes are included in organizational memory, and provide guidelines for future behavior. The police force and military represent good comparison industries in the sense that mistakes have a high legal cost and hiding and/or covering up problems can be a common strategy.

Employee silence in healthcare may simply reflect the social psychological need of individuals to identify with their organization. This is referred to as the “Abilene paradox,” which involves a common breakdown of group communication in which each member mistakenly believes that their own preferences are counter to the group’s and, therefore, does not raise objections (59). The Abilene Paradox is a desire not to “rock the boat.” So, how can we counter this desire? Obviously, leaders increasing psychological safety as a deterrent against employee silence should be the goal in the long-term, but the short to mid-term goals can include clinical leaders modeling desired behaviors such as humble inquiry, minimizing assumptions, and developing rapport (60). Employee silence is maintained at both the hospital board and senior management levels, in that the messages about work practices that compromise safety can be viewed as unwelcome. This results in perverse organizational dynamics—where people (i.e., clinical leaders as safety gatekeepers) are used as a means to an end, as tools and commodities rather than respected citizens (61). However, more contact between senior leaders and day-to-day operations at the ward level has the potential to reduce the gap between abstract policy and the reality of managing patient demands. Moreover, the emergence of kindness and compassionate leadership have the potential to create environments where information is shared earlier and more openly (62)—preventing larger problems that eventually need whistleblowers to illuminate them (63). The challenge for medical education is to figure out how it can stop healthcare professionals recycling the dysfunctional “rites of passage” behaviors that they have suffered under, if kindness and compassion are to be adopted.

## Data Availability Statement

The original contributions presented in the study are included in the article/supplementary material, further inquiries can be directed to the corresponding author.

## Author Contributions

AM and OL contributed equally to the conceptualization of the idea and contributed to writing the manuscript. Both authors contributed to the article and approved the submitted version.

## Conflict of Interest

The authors declare that the research was conducted in the absence of any commercial or financial relationships that could be construed as a potential conflict of interest.

## Publisher’s Note

All claims expressed in this article are solely those of the authors and do not necessarily represent those of their affiliated organizations, or those of the publisher, the editors and the reviewers. Any product that may be evaluated in this article, or claim that may be made by its manufacturer, is not guaranteed or endorsed by the publisher.
